# European Public Health News

**DOI:** 10.1093/eurpub/ckad033

**Published:** 2023-04-01

**Authors:** Marieke Verschuuren, Peter Geller, Laura T Murphy, Hanni Stoklosa, Jozef Bartovic, Hedinn Halldorsson, Marie Wolf, Isabel Yordi Aguirre, Anthony Staines, Floris Barnhoorn

**Affiliations:** EUPHA; EUPHA; EUPHA; EUPHA; EUPHA; EUPHA; EUPHA; EUPHA; (EPH Conference); (EPH Conference)

In this edition of the Public Health News, EUPHA’s executive director addresses the European Health Data Space and the promises it holds for (public) health research as well as the challenges that lie ahead during the development of this infrastructure. Geller *et al*. from WHO Regional Office from Europe look at human trafficking and the role of health workers in the identification, treatment and protection of trafficked persons and those at risk. Finally, Staines and Barnhoorn invite the readers to submit abstracts for the next European Public Health Conference in November 2023 in Dublin. The theme of this year’s conference is Our Food, Our Health, Our Earth: A Sustainable Future for Humanity.

## The European Health Data Space: a challenging but commendable endeavor

VerschuurenMarieke
EUPHA

One of the key elements of the European Health Union is the creation of the European Health Data Space (EHDS). The EHDS is a health-specific data sharing framework establishing clear rules, common standards and practices, infrastructures and a governance framework for the use of electronic health data by patients and for research, innovation, policy-making, patient safety, statistics or regulatory purposes. It is aimed to improve healthcare delivery across the EU, by empowering people to control their health data in their home country or in other EU countries; offer a consistent, secure, trustworthy and efficient framework for the secondary use of health data; and foster a genuine single market for digital health services and products.

As a science-based organization, EUPHA endorses the ambition to make health data more widely and efficiently available for statistics, research and policy-making. We therefore see the development of the EHDS as an important step towards better evidence for public health policy and practice. Clearly, the COVID-19 has shown both the importance of health information for policy-making and the current problems in health information systems, including data gaps, issues hampering timely access to existing data and problems with data linkage. This needs to be improved. In health information systems, generally, there tends to be a focus on the primary use of health data, i.e. the use of health data for the diagnosis and treatment of patients. That health data gathered in the process of care delivery should also be available for other (public) purposes, such as research and policy-making, often is overlooked in the design of health information systems. It is therefore laudable that the secondary use of health data (the HealthData@EU part of the EHDS) is taken into account in the EHDS proposal from the start, next to the primary use of health data (the MyHealth@EU part). Also, it is positive that the EHDS does not only cover electronic health record systems but also other sources such as research cohorts and survey data, which are also essential for population health monitoring and research.

The EHDS is a very ambitious project, which is likely to run into many different hurdles. Some of the main challenges relate to the many resources needed at member state and EU-level to develop, implement and maintain the data space; overcoming the current patchwork of systems, requirements, metadata standards, classifications, etc. used to ensure interoperability; the need for stringent and transparent safety procedures to protect the privacy of data subjects while at the same time not restricting the access to data so much that good quality research is hampered; and the need to engage with all relevant stakeholders including patients and citizens to build a transparent, purposeful and trustworthy construct.^1^^,^^2^ Thus, the development of the EHDS is not going to be an easy ride. But it is an extremely important one, which needs the input from and deserves the support of the public health community. EUPHA is proud to be part of the EHDS 2 pilot project consortium, that works on establishing the first building blocks of the infrastructure for sharing and analysing data between countries and testing it by means of several use cases.^3^ One might get discouraged by the daunting task ahead, but I believe that when we join forces, more and better data for health research can be achieved, leading to better evidence-informed health policy-making, and ultimately, improved health for European citizens.

## From reactive to proactive: the role of health care in fighting human trafficking

GellerPeter
EUPHA
MurphyLaura T
EUPHA
StoklosaHanni
EUPHA
BartovicJozef
EUPHA
HalldorssonHedinn
EUPHA
WolfMarie
EUPHA
Yordi AguirreIsabel
EUPHA

Human trafficking is a lucrative crime and a violation of human rights that exploits women, children and men—and front-line health workers are often the only public servants that trafficked people meet during their ordeal. What is staggering is that an example study from the USA has shown that more than half of the trafficked remain unidentified by the health workers treating them, and that health workers often feel that they lack the knowledge and training on how to identify and treat individuals trapped in trafficking and its survivors.

While all WHO European Region Member States have ratified the Palermo Protocol that recognizes human trafficking as a criminal enterprise and a violation of human rights, many of them have yet to implement National Referral Mechanisms that create state-wide protocols. Indeed, most health systems in the WHO European Region—consisting of 53 Member States—have no specific policies on the matter, and lack the capacity to identify and treat trafficked persons. According to the latest findings, in 2020, the first year of the COVID-19 pandemic, nearly 20 000 trafficking cases were reported in Europe and Central Asia. That figure is likely to be both a vast underestimate, as well as to have grown drastically in light of the war in Ukraine and COVID-19, since emergencies heighten the socioeconomic risk factors for trafficking, while temporarily jolting health systems.

Human trafficking is the recruitment, transportation, transfer, harbouring or receipt of people through force, fraud or deception. People are trafficked for many different forms of exploitation such as forced labour, sexual exploitation and forced begging. It affects people of all ages, races, sexes, nationalities, ethnicities, religions, gender identities, sexual orientations and abilities, regardless of national economy, rates of migration or gender equality legislation. Contrary to what many believe, trafficking can be domestic as well as cross-border.

The severe health effects of trafficking and the health needs of trafficked individuals are well-documented. Trafficking has wide-ranging impacts on people’s physical and mental health. Those subjected to it need a variety of services, from the treatment of injuries, sexual and reproductive healthcare, to psychosocial support including counselling for conditions such as post-traumatic stress disorder or depression.

As the first UN report of its kind, based on a scoping review of literature published between 2015 and 2022, a new report by the WHO Regional Office for Europe provides policymakers with an overview of the evidence on the untapped potential of health systems to address trafficking, as well as concrete steps to prevent and respond to it. The report calls on Member States to take decisive action on this all too often ignored intersection of healthcare and trafficking, and establish intersectoral policy and advocacy to prevent and respond through health systems. The body of research repeatedly suggests that the primary barriers to healthcare access for trafficked individuals include a sense of ‘disenfranchisement’, shame, distrust, lack of health insurance or identification and ignorance of rights. Every one of these barriers can be addressed through a public health response. At the centre of any anti-trafficking policy, is universal health coverage, meaning universal, affordable access to health services. Any national plan must also include both prevention and response. The root causes of trafficking need to be addressed: that is social determinants of health such as gender inequalities, gender-based discrimination, poverty, sexual abuse, homelessness and socioeconomic marginalization.

WHO has identified a number of policy considerations. Health systems and medical schools need to significantly increase investment to strengthen capacity and sensitize health workers. Quality of care should always be prioritized over identification. Prevention should be rights-based and survivor-centred, focused on both building resilience and mitigating risk to patients. Everyone should have universal, low-barrier access to health care, regardless of status and resources. Health systems should address structural barriers to healthcare access, which include understaffing, underfinancing and lack of health workers’ knowledge. Health systems need a ‘no wrong door’ policy that identifies and treats trafficked individuals at a variety of healthcare settings. They should systematically develop training for all staff that addresses provider unconscious bias and that is based around survivor-centred and trauma-informed care.

It is time we took a proactive rather than merely a reactive stance to human trafficking. Our health systems have a responsibility to promote and protect the health and rights of trafficked people. We should seize the unique opportunity that health workers have—to identify, treat and protect trafficked persons and those at risk.

## Our Food, Our Health, Our Earth: A Sustainable Future for Humanity

StainesAnthony
(EPH Conference)
BarnhoornFloris
(EPH Conference)

16th European Public Health Conference

8–11 November 2023

Convention Centre Dublin (The CCD), Dublin, Ireland

### Abstract submission for Dublin 2023

EPH Conferences offer excellent opportunities to learn from the latest research and practice, to network with experts and colleagues and to expand your professional horizons. Why don’t you participate at Dublin 2023 by sharing your work with colleagues? Abstracts are invited for workshops and single presentations in all areas of public health research, practice, policy and education. Abstract submission is open from 1 February to 1 May 2023. More information.

In collaboration with EUPHAnxt, we provide an Abstract Tutoring Programme offering an opportunity for young and/or less experienced abstract submitters to receive feedback from experienced reviewers. The programme is especially targeted at researchers who have limited access to colleagues to ask for guidance and comments on their proposed abstracts. More information.

EPH Conference offers prizes for the best single abstract. The prize is determined by the scoring of the EPH Conference International Scientific Committee. The winner will be awarded a certificate and waiver of registration fees for the EPH Conference 2024. Details can be found here.

### Our Food, Our Health, Our Earth: A Sustainable Future for Humanity

Over the next decade, choices will be made which will determine the future of our advanced technological civilisation. We now have no way out in terms of climate emergency. The global climate is rapidly deteriorating. We know what to do, to deliver a future for our society, but it’s less clear that we will do it. COP27 achieved little in the ambition to keep the 1.5 degree limit alive and pull humanity back from the climate cliff. Building a future, any kind of future, for us and our children, demands a new attention to sustainability. The EPH Conference in Dublin hopes to open part of this discussion, with a focus on sustainability, food and health. We will look specifically at global health, the use of digital tools, equity in health and the necessary staffing and skills to provide good care. We will, in line with the overall conference theme, look in depth at the climate emergency, and very specifically at the human food supply.

We look forward to seeing you all in Dublin. The city is well known as a literary city, but its long history of public health in Ireland is less well known. These intersect strikingly in one man -William Wilde, society doctor, hospital founder and father of Oscar, who needs no introduction, and Willy, who was an alcoholic and a journalist. He wrote extensively on eye surgery, Irish antiquities, Irish folklore and on the health of the Irish population. You will have a chance to discover his city, in all its brightness and darkness, as well as learning something more about making our futures.

We look forward to seeing you all in person, in Dublin.
